# N-methyl-D-aspartate receptor antibody-mediated neurological disease: results of a UK-based surveillance study in children

**DOI:** 10.1136/archdischild-2014-306795

**Published:** 2015-01-30

**Authors:** Sukhvir Wright, Yael Hacohen, Leslie Jacobson, Shakti Agrawal, Rajat Gupta, Sunny Philip, Martin Smith, Ming Lim, Evangeline Wassmer, Angela Vincent

**Affiliations:** 1 Nuffield Department of Clinical Neurosciences, John Radcliffe Hospital, University of Oxford, Oxford, UK; 2 Department of Paediatric Neurology, Birmingham Children's Hospital, Birmingham, UK; 3 Children's Neurosciences, Evelina Children's Hospital @ Guy's and St Thomas’ NHS Foundation Trust, King's Health Partners Academic Health Science Centre, London, UK

**Keywords:** Encephalitis, Autoantibody, NMDA receptors, immunotherapy, Neurology

## Abstract

**Objective:**

N-methyl-D-aspartate receptor antibody (NMDAR-Ab) encephalitis is a well-recognised clinico-immunological syndrome that presents with neuropsychiatric symptoms cognitive decline, movement disorder and seizures. This study reports the clinical features, management and neurological outcomes of paediatric NMDAR-Ab-mediated neurological disease in the UK.

**Design:**

A prospective surveillance study. Children with NMDAR-Ab-mediated neurological diseases were voluntarily reported to the British Neurological Surveillance Unit (BPNSU) from November 2010 to December 2011. Initial and follow-up questionnaires were sent out to physicians.

**Results:**

Thirty-one children fulfilled the criteria for the study. Eight presented during the study period giving an incidence of 0.85 per million children per year (95% CI 0.64 to 1.06); 23 cases were historical. Behavioural change and neuropsychiatric features were present in 90% of patients, and seizures and movement disorders both in 67%. Typical NMDAR-Ab encephalitis was reported in 24 children and partial phenotype without encephalopathy in seven, including predominantly psychiatric (four) and movement disorder (three). All patients received steroids, 22 (71%) received intravenous immunoglobulin, 9 (29%) received plasma exchange,and 10 (32%) received second-line immunotherapy. Of the 23 patients who were diagnosed early, 18 (78%) made a full recovery compared with only 1 of 8 (13%) of the late diagnosed patients (p=0.002, Fisher's exact test). Seven patients relapsed, with four needing additional second-line immunotherapy.

**Conclusions:**

Paediatric NMDAR-Ab-mediated neurological disease appears to be similar to adult NMDAR-Ab encephalitis, but some presented with a partial phenotype. Early treatment was associated with a quick and often full recovery.

**What is already known on this topic?**

- Autoimmune encephalitis is increasingly recognised as an important cause of encephalitis in adults and children.
- Paediatric N-methyl-D-aspartate receptor-antibody (NMDAR-Ab) encephalitis is a complex multisymptom disease, but treatable with immunotherapy.

**What this study adds?**

- Paediatric NMDAR-Ab encephalitis can present with a single clinical feature predominating.
- Plasma exchange in the early stages of disease may be associated with a quicker recovery to a premorbid level of functioning.
- Most patients, particularly those diagnosed and treated early, make a full recovery, and this should be the aim of therapy.

## Introduction

N-methyl-D-aspartate receptor antibody (NMDAR-Ab) encephalitis is the most widely studied of the recently described autoimmune encephalitidies.^1^ ^2^ Primarily affecting young adults and children, the typical presentation is with subacute onset behavioural change, neuropsychiatric features and seizures, usually progressing to movement disorder, hypoventilation, reduced consciousness and autonomic instability.^3^ The association with an underlying ovarian teratoma^4^ depends on age and sex, and is most frequent (up to 50%) in young women.^5^ ^6^ The paediatric presentation has been described as more ‘neurological’ than the more psychiatric presentation in adults.^6^ ^7^

Patients are treated with tumour resection if required, first-line immunotherapy (intravenous and/or oral steroids, intravenous immunoglobulin, and/or plasma exchange (PLEX)) and second-line immunotherapy (cyclophosphamide or rituximab) if indicated.^4^ More than 75% of all patients have a substantial recovery, with early recognition and treatment predictive of a good outcome.^4^ This information, however, has been largely gathered from patient cohorts, mainly comprising retrospective data,^6^ ^7^ and as yet, no incidence rates and outcomes have been reported from population-based prospective cohorts. Here, we report a prospective surveillance study in the UK to ascertain incidence, clinical, investigative features and outcomes of childhood (age <18 years) NMDAR-Ab encephalitis.

## Method

### Study design

A UK-wide prospective surveillance study of NMDAR-Ab encephalitis in children (1–17 years 11 months), in conjunction with the British Paediatric Neurology Surveillance Unit (BPNSU), recruited patients from November 2010 to December 2011 (13 months). Through a web-based portal (http://www.bpnsu.co.uk/), monthly notification emails were sent to all registered consultant paediatric neurologists during the study period. Clinicians replied to the email notifying any cases or confirming ‘nothing to report’. Upon receipt of a positive notification, the surveillance unit provided the investigating team with a BPNSU case number and clinician contact details.

### Case definition and identification

The case definition for this study was ‘any child or young adult, who presents with new onset of acute behavioural change, seizures, dystonias or dyskinesias and with antibodies to the NR1 subunit of the NMDAR in the serum and/or CSF’. Clinicians were asked to report both new and previous cases. The study team contacted the clinician directly and sent two questionnaires: one at notification and one at 12 months (see online supplementary information). ‘Late diagnosis’ was defined as identification of NMDAR-Abs >6 months from disease presentation; 19 of these cases have been reported previously as part of a case series, cohort or as case reports.^8–10^ Treatment response was derived from the clinician responses in the questionnaire, and mRS (modified Rankin Scale) for children (appended to the follow-up questionnaire) was used to measure outcomes.

### Statistical analysis

Descriptive statistics were used to summarise the key components of the dataset. Fisher's exact test (two-tailed) was used to compare clinical details in GraphPad Prism V.6.

### Approvals

The study proposal was approved by the BPNSU executive committees. The study had approval from the UK Multicentre Research Ethics Committee and the Oxfordshire Regional Ethical Committee A (07/Q1604/28) with a substantial amendment (1) approved on 30 April 2010.

## Results

Over the study period (13 months), 1526 email responses were received from 171 clinicians reporting 35 known and 10 new cases. A review of the Oxford neuroimmunology database confirmed the positive NMDAR-Ab results. Three children with positive results were identified from the Oxford database and not reported to the BPNSU. Of the 35 completed questionnaires received, 31 cases from 13 different centres met the case study definition. Seven of these patients had been diagnosed late (over 6 months). Four patients were excluded; one was too old at the time of presentation (19 years old); the second patient was excluded as his NMDAR-Ab positivity was low and his serum immunoglobulin (Ig)G also bound to other antigens suggesting non-specificity, possibly due to previous intravenous immunoglobulin (IVIG) treatment causing raised serum IgG levels; two further cases were excluded as they did not fulfil the clinical criteria of the case definition. Both these patients presented with white-matter disease now known to be associated with NMDAR-Abs.^11^ These cases and cases from other centres have been reported separately.^12^ The remaining 24 cases were diagnosed within 8 weeks of symptoms onset, but only eight within the study period.

### Demographics

**Figure 1:**
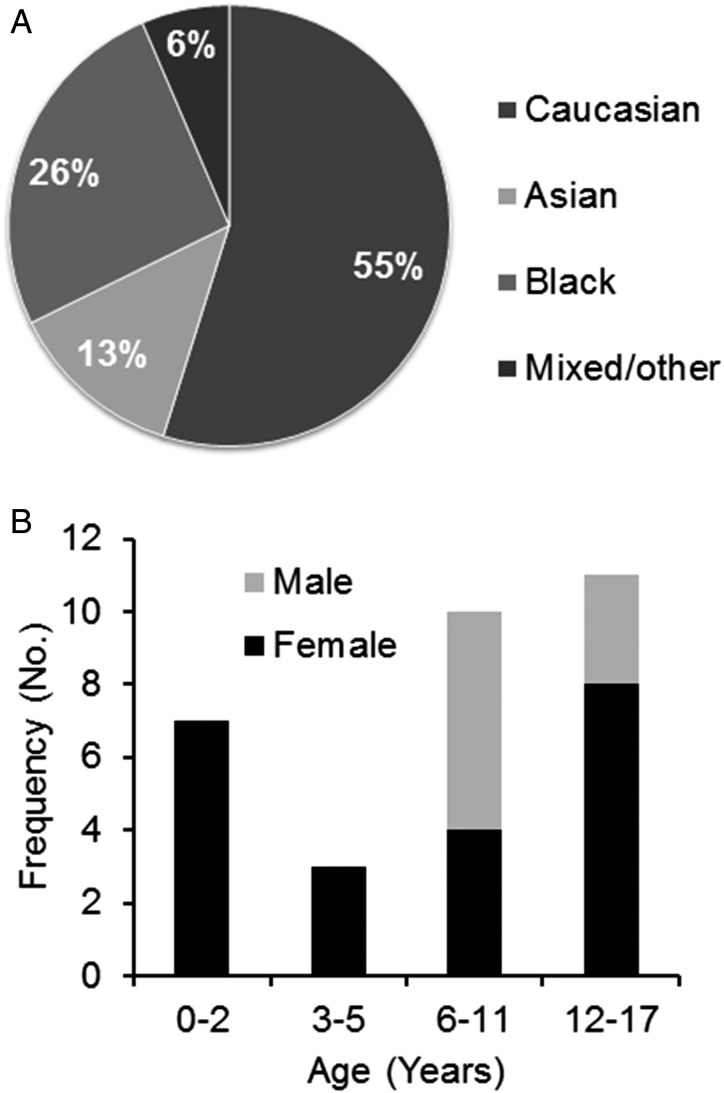
Demographic data of UK patients with N-methyl-D-aspartate receptor-antibody encephalitis. Age of onset stratified to sex (A) showing an overall female predominance. Boys were more likely to present at over the age of 5 years (p=0.03, Fisher's exact test)**.** Ethnicity of the patients (B), 14 patients (45%) were non-Caucasian.

Patients’ demographics are described in figure 1. The majority of patients were female (23/31; 74%) with a median age of 8 years (range 22 months–17 years). Male patients, despite the small numbers, were older (median 11 years; range 6–17 years; p=0.03); 55% of the patients were Caucasian (figure 1). There were eight patients newly diagnosed over 13 months giving an estimate of incidence, based on a paediatric population of 12 million in the UK (http://www.ons.gov.uk), of 0.85 per million children per year (95% CI 0.64 to 1.06).

### Clinical features of all patients

Good premorbid health and development were seen in 28/31(90%); two patients had an uncomplicated Tetralogy of Fallot repair in infancy and another patient had a XXX karyotype with developmental delay. There was no significant family history of autoimmune disease.

The clinical features are summarised for each patient in table 1A, B. Behavioural change and/or neuropsychiatric features were the initial presenting symptom in 90% (28/31). During the course of the disease, seizures and new onset movement disorders were both seen in 21/31 patients (68% in each case). Sleep dysfunction was reported in 16/31 (52%) and autonomic features were found in 12 (39%) cases, with cardiac abnormalities including one cardiac arrest, and hypoventilation requiring intensive care support in three cases. Individual patients exhibited myoclonus, neuromyotonia or transient limb weakness. Partial phenotypes, predominant movement (n=3) or psychiatric features (n=4), were reported more frequently in boys (n=4) (4/8 boys vs 3/23 girls, p=0.05, Fisher's exact test). The patients with partial phenotypes had at least one additional symptom (behavioural change, movement disorder, psychiatric features or seizures) and were not monosymptomatic (table 1).

**Table 1:** Predominant symptoms at presentation, and outcomes at final follow-up

(A)					
Case number	Age: sex	Clinical features	Time to diagnosis/time to treatment response	Treatment group	Outcome/ΔmRS score*
2†	14: F	BC, S, MD, Sp	6 months/2 days	A	Full recovery no sequelae/(5)
3	F	BC, S, MD	9 months/90 days	D	Cognitive problems/(3)
4	10: F	BC, S	>1 year/no response	A	Cognitive problems/(1)
16	6: M	BC, S, MD	>1 year/7 days	A	Seizures, on treatment/(4)
20	10: F	BC, S, MD	>1 year/no response	A	Significant disability, relapse, leucoencephalopathy on MRI (0)
28	8: M	BC, S, MD	7 months/3 days	A	One encephalopathic relapse treated with steroids/(2)
*Partial phenotypes*					
19	10: F	MD, Sp	> 1 yr/28 days	A	Symptom recurrence when unwell (1)

(B)					
Case number	Age: sex	Clinical features	Time to diagnosis/time to treatment response	Treatment group	Outcome/ΔmRS score†

1	14: F	BC, S, MD	1 month/90 days	D	Full recovery no sequelae/(3)
5	3: F	BC, S	1 week/7 days	A	Full recovery no sequelae/(4)
6	5: F	BC, S, MD	1 month/14 days	C	Antibodies remain high, cognitive problems/(3)
7	9: M	BC, S(EPC),MD	2 months/90 days	A	Full recovery no sequelae/(3)
9	2: F	BC, S, MD, Sp	5 weeks/7 days	A	Full recovery no sequelae/(4)
10	14: F	BC, S, MD,	1 week/29 days	D	One relapse treated with IV/PO steroids and MMF, full eventual recovery no sequelae/(5)
11	2: F	BC, S, MD	<1 week/72 days	D	Full recovery no sequelae/(4)
12	8: F	BC, S, MD	2 months/60 days	C	Full recovery no sequelae/(3)
13	15: F	BC, S, MD, Sp		A	Lost to follow-up
14	13: F	BC, S, MD, Sp, NMT	1 week/14 days	A	Relapse at 1 year treated with AZT and MMF, full eventual recovery/(2)
15	2: F	BC, S, MD	2 weeks/30 days	A	Ongoing seizures, cognitive problems, antibodies elevated/(3)
17	4: F	BC, S, MD	2 weeks/30 days	A	Developmental arrest same as predisease stage, cognitive and behavioural problems (3)
22	17: F	BC, S, MD	3 weeks/3 days	B	Full recovery no sequelae/(5)
24	14: F	BC, S, MD	1 month/	A	Cognitive problems and seizures/(2)
26	3: F	BC, S, MD	1 month/28 days	D	Full recovery no sequelae/(1)
27	11: M	BC, MD	12 days/8 days	A	Full recovery no sequelae/(5)
30	2: F	BC, MD	2 weeks/19 days	A	Cognitive and behavioural problems/(2)
31	2: F	BC, MD	4 weeks/67 days	D	Full recovery no sequelae/(5)
*Partial phenotypes*					
8	17: M	NPsych, BC, S	3 weeks/14 days	B	Full recovery no sequelae/(4)
18	5: F	MD, S, Sp	<1 week/7 days	A	Full recovery no sequelae/(3)
21	12: M	MD, NPsych	8 weeks/7 days	A	Full recovery no sequelae/(1)
23	16: M	NPsych , BC	1 month/7 days	C	Two relapses before MMF, none since started (1)
25	15: F	NPsych, BC	2 months/2 days	D	Relapse treated with steroids, cyclophosphamide and rituximab (4)
29	14: M	NPsych, BC	2 months/5 days	A	Relapse treated with PLEX and second-line immunotherapy; eventual full recovery (0)

(A) lists patients with late diagnosis; (B) lists patients diagnosed within 8 weeks from symptoms onset. Patients with partial phenotypes were defined based on a lack of encephalopathy as evaluated by clinician.

*Outcome at final follow-up. ΔmRS score from nadir to 12-month postpresentation.

†Denotes patient with ovarian teratoma.

AZT, azathioprine; BC, behavioural change; EPC, epilepsia partialis continua; IV/PO, intravenous/oral; MD, movement disorder; MMF, mycophenolate mofetil; NMT, neuromyotonia; NPsych, neuropsychiatric; PLEX, plasma exchange; S, seizures; Sp, speech dysfunction.

### Paraclinical features of all patients

Cerebrospinal fluid (CSF) was abnormal with pleocytosis in 45% (13/29). Eight of 12 (67%) had intrathecal oligoclonal bands. Seven serum/CSF pairs were positive for NMDAR-Abs; two CSFs sent later during the disease course were negative. MRI scans were normal in 20/31 (65%), but high signal changes in cortical (frontal, frontoparietal, temporal, hippocampi), and subcortical areas (basal ganglia) were seen in 7/31 (23%). Electroencephalography (EEG) was encephalopathic in the majority (28/30; 93%). The finding of extreme delta brush was not reported. The EEG was normal in two of the cases with partial phenotypes (Cases 19 and 9). Three patients had a positive antistreptolysin O titre, one of whom was also positive for IgM Epstein–Barr virus antibodies. Other antibodies identified included antinuclear antibody and extractable nuclear antigen in one patient, antibasal ganglia antibodies and voltage-gated potassium channel-complex antibodies (661 pmol/L) in another.

### Clinical response to initial therapy: speed and extent

**Figure 2:**
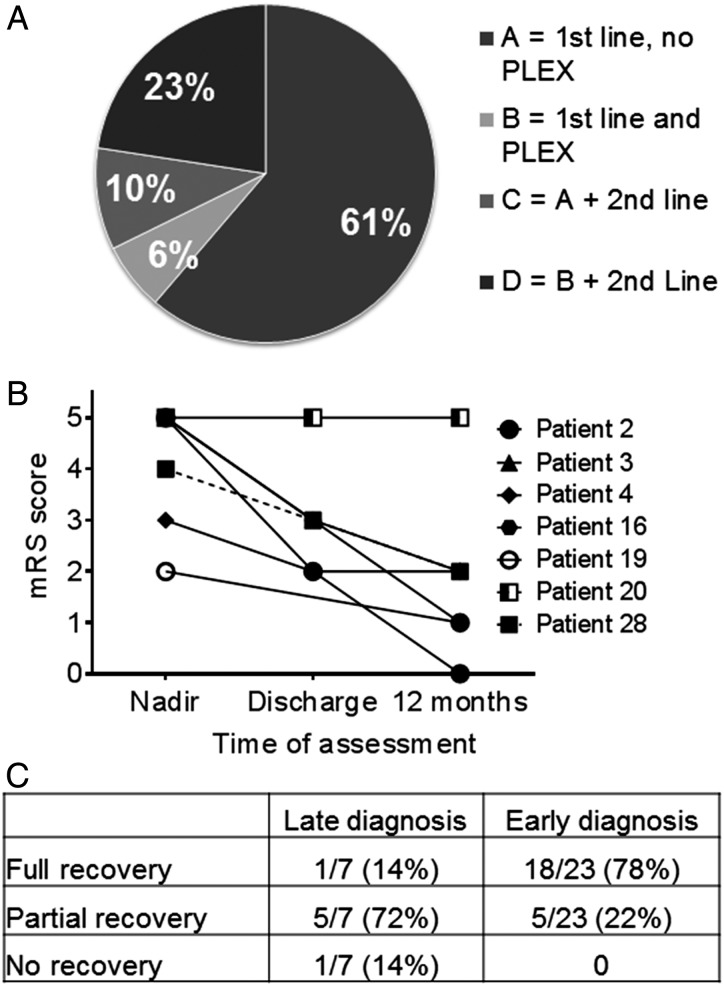
(A) Pie chart showing the four treatment groups of the whole cohort. Group A had intravenous immunoglobulin and oral/ intravenous steroids only (first-line) and no plasma exchange (PLEX); Group B had first-line immunotherapy and plasma exchange; Group C had first-line immunotherapy, no PLEX and second-line immunotherapy; Group D had all the treatments (first-line, PLEX and second-line immunotherapy). (B) Graph to show the late diagnosed patient group continues to make progress over time despite no aggressive or long-term treatment. (C) Table summarises outcomes showing that all patients diagnosed early make a full/partial recovery (p=0.0011**, χ^2^ for trend).

**Figure 3:**
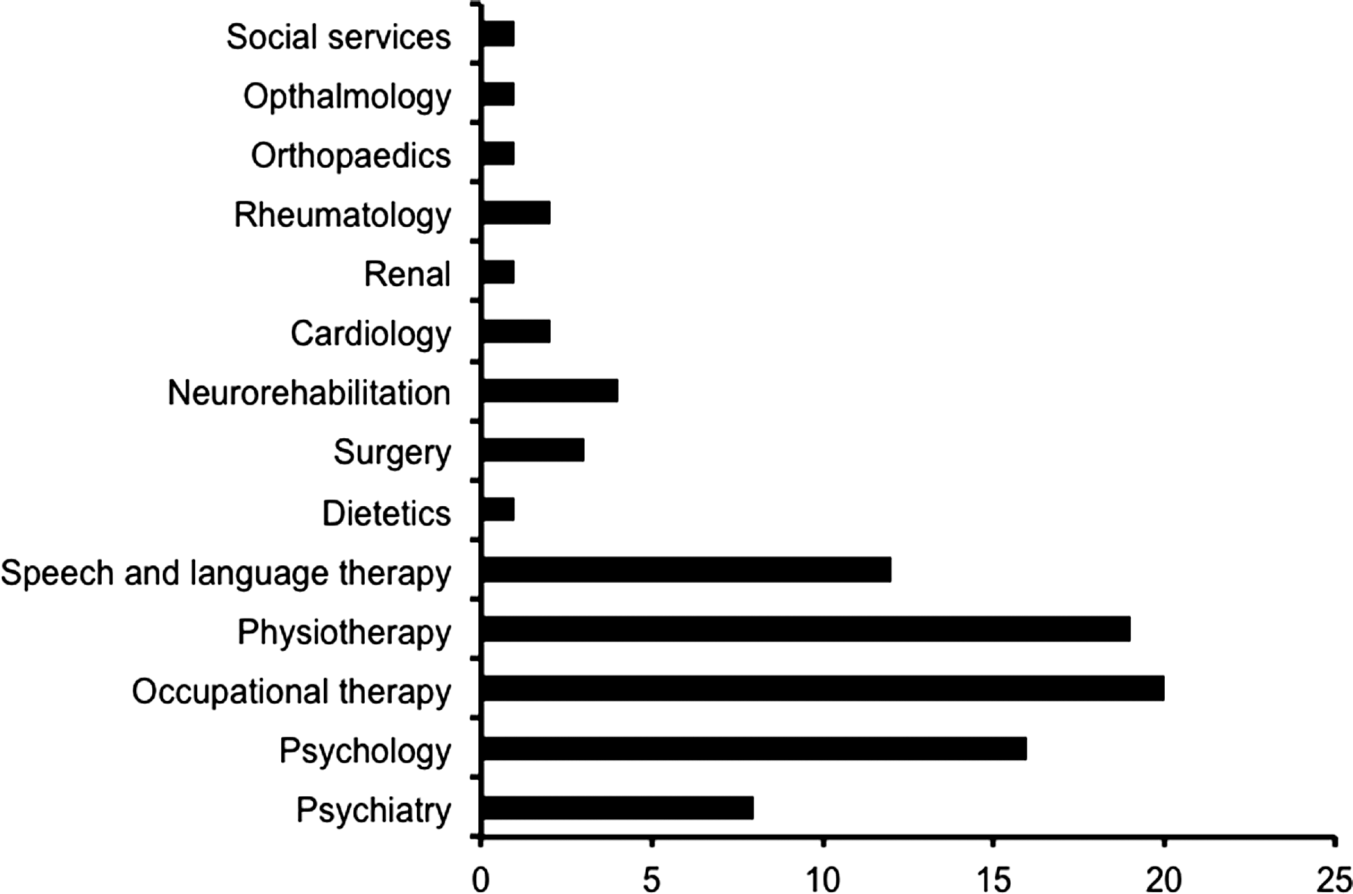
Bar chart to show number and range of allied health professionals and other medical specialities required in the management of N-methyl-D-aspartate receptor-antibody neurological disease in paediatric patients in the UK.

All patients received steroids; 22 (71%) received IVIG, 9 (29%) received PLEX and 10 (32%) received second-line immunotherapy. Four treatment groups were identified within the whole cohort of 31 patients (figure 2A). The most frequently used (61%) were IVIG and steroids (Group A); PLEX was added to these in two patients (Group B). Three patients had IVIG, steroids, no PLEX, but second-line immunotherapy (Group C). Seven patients had steroids, IVIG, PLEX and second-line immunotherapy (Group D). A total of 10 (32%) patients were treated with second-line or long-term immunotherapy using cyclophosphamide (n=3), rituximab (n=3) or both (n=3), with mycophenolate mofetil (MMF) initiated in one patient. No significant treatment complications/side effects were reported in any of the 31 patients. Eighteen of 31 (58%) patients showed a response to immunotherapy within 30 days, but in three patients, this took up to 90 days. Thirteen patients required intensive care support during their illness (13/31; 42%). The median length of patient stay in hospital was 60 days (range 10–365 days), and four (range 1 day–7 days) other health/medical professionals were needed during admission for each case (figure 3). There was no correlation between age of presentation and response to immunotherapy, and no relationship between initial response to immunotherapy and outcome at 12 months (table 1). However, 89% (8/9) patients who received PLEX during their initial treatment made a full eventual recovery compared with 47% receiving IVIG and steroids only (p=0.049, Fisher's exact test), although this might have been confounded by additional second-line immunotherapies, as 7/9 of the patients had also received second-line immunotherapy.

### Comparison of clinical response between early and late diagnosis groups

#### Late diagnosis (>6 months)

Seven patients were not tested for NMDAR-Abs until over 6 months from symptom onset (table 1A). Despite the delay in diagnosis, all had received steroids and/or IVIG, and one had MMF (Case 3). One child (Case 2), who responded to first-line immunotherapy, was found to have NMDAR-Abs 6 months after initial presentation, while asymptomatic and off treatment, and was consequently found to have an ovarian teratoma. The teratoma was removed and the patient remained asymptomatic with no relapses. The remaining six patients still suffer from ongoing behavioural, cognitive problems and seizures up to 5 years from initial presentation, and one child (Case 20) was intractable to all treatment. The median mRS score at nadir was 5 (range 2–5), and 2 (range 0–5) at 1-year follow-up. However, six patients continued to make progress postdischarge, despite limited or no ongoing treatment (figure 2B).

#### Early diagnosis (within 8 weeks)

Twenty children (65%) were diagnosed within 8 weeks of symptom onset, with a median time of 3 weeks from presentation to diagnosis (no patients were diagnosed between 8 weeks and 6 months). The clinical details are summarised in table 1B. The seven patients with partial phenotypes (Cases 8, 18, 19, 21, 23, 25, 29) were diagnosed 3 weeks later than the patients presenting with full NMDAR-Ab encephalitis phenotypes. At last follow-up (12–60 months), 78% (18/23) of patients with early diagnosis had made a full recovery with no sequelae and were rated an mRS score of 0 compared with the late diagnosed group where only one patient (Case 2) made a full eventual recovery (figure 2C, p=0.0011, Fisher's exact test). The median mRS score at nadir was 4 (range 2–5), and 1 (range 0–4) at 1-year follow-up. Eight early diagnosed patients, seven with typical features of NMDAR-Ab encephalitis and one predominantly neuropsychiatric, made a full recovery within 12 months. Five of these eight patients (63%), received PLEX as part of their initial treatment. Out of the four others who received PLEX, three also made a full recovery. Five of 23 (22%) patients had not made a full recovery with ongoing cognitive problems and learning difficulties, and one has refractory seizures requiring treatment with multiple anti-epileptic drugs (AEDs) (Case 15) (table 1A, B). Four out of these five received only steroids and IVIG as treatment.

### Relapses

Seven patients (23%) had a clinical relapse, which was also confirmed by further antibody testing, (Cases 10, 14, 20, 23, 25, 28, 29); six out of seven relapsing patients (86%) had received steroids and IVIG only as their initial treatment. The median time to relapse from original presentation was 12.5 months (range: 2–60 months). The clinical presentation at the time of relapse was similar to acute but milder in severity. All relapses were treated with first-line immunotherapy with additional PLEX in one. Four of the seven (57%) patients went on to receive second-line or long-term immunotherapy at the time of relapse, with no further relapse, and full recovery in three patients. Only one patient had more than one relapse (Case 23).

## Discussion

NMDAR-Ab-associated neurological disorders are now well recognised in children who represent around 40% of the total number of cases.^5^ ^6^ As most studies were done retrospectively on patients with well-defined NMDAR-Ab encephalitis,^5^ ^7^ the incidence of this condition and the existence of less typical phenotypes has not been clear. In this prospective study, we gathered details on eight NMDAR-Ab-positive children presenting in the UK between November 2010 and December 2011, and an additional 23 who had already been identified as positive for serum NMDAR-Abs. From our data, we estimate the incidence to be 0.85/million children/year. The BPNSU relies on voluntary reporting by clinicians and, hence, could result in underestimation of the true incidence.

Our cohort had demographics similar to previously reported cohorts with female predominance and only 55% Caucasians.^3^ ^5^ ^6^ Only one girl had an ovarian teratoma. By contrast with a previously reported paediatric case series suggesting that children are more likely to present with a neurological rather than psychiatric phenotype,^7^ behavioural change and/or neuropsychiatric symptoms were reported in 90% of children. Nine patients (31%) presented under the age of 3 years confirming recent reports, and highlighting the importance of testing for NMDAR-Ab even in very young children.^13^

We did not identify a purely monosymptomatic group, but some patients (partial phenotypes) were not encephalopathic. These patients had additional associated features (particularly cognitive) in keeping with symptoms seen with NMDAR hypofunction. Although these were subtle, their identification was important in managing the patients appropriately and screening for the associated neoplasms. It remains unclear whether the treatment prevented these patients from developing full-blown encephalopathy, but the time from symptom onset to treatment in patients with partial phenotypes was longer than in patients presenting with NMDAR-Ab encephalitis. The psychiatric/movement disorder predominant disease was seen more frequently in male patients, in contrast with the overall female predominance. These phenotypical differences between the sexes were recently reported in an adult study^14^ suggesting that hormonal influences on the immune system or on target neurons, may contribute to the different clinical pictures.

The paraclinical features of our patients were similar to the previously reported series^5^ ^7^ with normal MRI brain, encephalopathic EEG with or without epileptiform discharges, and CSF abnormalities in 45%. The pattern of extreme delta brush was not reported, although the neurophysiologists would not have looked for this at the time of the questionnaire completion.^15^ Positive viral serology was seen in two of the patients; the association between NMDAR-Ab encephalitis and some viruses, particularly herpes simplex virus (HSV) has previously been reported by us^16^ and others,^17–19^ with either co-occurrence at onset or HSV encephalitis triggering NMDAR-Ab encephalitis.

All patients received immunotherapy but with different regimes reflecting institutional or clinician preference. Most patients (78%) diagnosed and treated early made a full recovery compared with those diagnosed late (>6 months). There remains no controlled trial to provide high-quality evidence on the optimal therapeutic strategy for NMDAR-Ab encephalitis, nor is there the definitive distinction of what constitutes first-line and second-line therapy. A large cohort analysis^6^ appears to suggest the additional benefits of second-line therapy (rituximab, cyclophosphamide), when first-line therapy fails (steroids, IVIG and PLEX). Certainly, in the current study, the more symptomatic patients and those who relapsed were more likely to have their treatment escalated in this way. Although we did not find a difference in the long-term outcome between patients who received second-line immunotherapy or not, full recovery was seen in nearly all (8/9) patients receiving PLEX; this is higher than the group which only received steroids and immunoglobulin (p=0.049), and also higher than the reported literature.^5^ ^6^ However, these data are confounded by early treatment and second-line immunotherapy in seven of the nine (78%) patients, in keeping with a recent report suggesting that the use of rituximab in paediatric neuroimmune conditions was associated with greater change in mRS in patients given rituximab early in their disease course compared with those treated later.^20^ A previous study in adults demonstrated a better cognitive outcome in patients treated early.^21^ Nevertheless, the response to treatment seen, even in the patients who were diagnosed late, support the role of any form of immunotherapy in all stages of active disease.

The average time of hospital stay in our cohort was 60 days, higher than in other forms of acquired encephalopathies,^22^ with 42% requiring admission to intensive care units. Appropriate management of these patients is challenging and frequently requires input of a multidisciplinary team. Speech and language therapists, physiotherapists and occupational therapists form the mainstay of this fundamental support team and should be engaged early in disease presentation for maximal benefit to the patients.

Taken together, careful use of treatment regimes and supportive therapies are essential to maximise patient care in this difficult-to-treat condition. Nevertheless, our study suggests that complete recovery can be achieved in the majority of paediatric NMDAR-Ab-mediated neurological diseases. Further studies of larger prospective cohorts and randomised treatment trials are still needed to establish the role of second-line immunotherapy and the duration of treatment needed for this condition.

## Supplementary Material

Web supplement
