# Supplementary material for: N-methyl-D-aspartate receptor antibody-mediated neurological disease: results of a UK-based surveillance study in children
Source: Arch Dis Child. 2015 Jan 30;100(6):521–6. doi: 10.1136/archdischild-2014-306795 (PMC4453622; doi:10.1136/archdischild-2014-306795)
Supplement: Web supplement [file archdischild-2014-306795-s1.pdf]

QUESTIONNAIRE

Details of clinician completing form:
Name:
Hospital:
Telephone:
Email:
Date completed:

Patient information:
Age: Sex: Ethnic origin:
Date of admission to hospital:

Presenting symptoms – please circle if applicable and specify day of illness when symptom first appeared

Neuropsychiatric
Acute confusion Agitation Behavioural change Speech dysfunction
Seizures focal/generalised Other/Details____

Movement abnormalities
Orolinguofacial dyskinesias Choreoathetosis Dystonia Muscle rigidity
Sterotyped movements Other/Details____

Autonomic instability
Tachycardia Enuresis Sleep dysfunction Hypertension
Hyperthermia Hypoventilation Other/Details____

Investigations

	Performed	Normal	Abnormal (please give details or attach formal report)
MRI			
EEG			
CSF			
Abdominal imaging			
Other investigations<br>*<br>*			

Tumour present? Yes/No What tumour? ____________________________________

Treatment

Treatment	Received?	Drug name, dose, duration of therapy
Plasmapharesis		
Intravenous immunoglobulin		
Intravenous steroids		
Oral steroids		
Other immunotherapy		
Antivirals / Antibiotics		

Did the child need intensive care during the illness? If yes, then why?___________________________________
____

Medical history:
Any previous medical history?
Any family history of autoimmune disease?

Thank you for completing this questionnaire.

Please return to Dr Sukhvir Wright (c/o Professor Angela Vincent), Neurosciences Laboratory, West Wing Level 5, John Radcliffe Hospital, Oxford. OX3 9DS. Fax: 01865 234320 Email: sukhvir.wright@nhs.net

FOLLOW-UP QUESTIONNAIRE
BPNSU study of NMDAR-Ab encephalitis patients

Details of clinician completing form: **Patient study ID:**

Name:
Hospital:
Telephone:
Email:
Date completed:

How many days/months between 1) SYMPTOM ONSET AND DIAGNOSIS?..
2) SYMPTOM ONSET AND FIRST SIGN OF RECOVERY?........................
3) START OF IMMUNOTHERAPY AND RECOVERY?..............................

Total length of hospital stay ☐ days

Involvement of allied health professionals/other medical teams in treatment (fill table below)

Physiotherapy	OT	Psychiatry	Psychology	Surgery	Neurorehab	Others (please list)
Y/N	Y/N	Y/N	Y/N	Y/N		

Outcome of initial illness
At 3 time points to record the following (both scoring scales, MRS* and PCPC**, overleaf);

Days from onset (D)	D= nadir of illness		D= discharge from hospital		D= 1 year follow-up	
Treatment						
Score	MRS	PCPC	MRS	PCPC	MRS	PCPC
Residual features, e.g. seizures, cognitive problems						

Relapses Y/N
If Y to above:

Relapse event number	Time from onset of disease to relapse	Presenting features of relapse	Investigations during relapse *MRI,EEG, USS CSF/SERUM NMDAR-Ab*	Treatment required and response to treatment	Neurological and cognitive outcome post relapse
1					
2					

Any additional information regarding relapse? *e.g. possible contributory factors, lack of overall improvement, parental anxiety.*

Patient assessment at last follow-up. *Please include time seen after initial diagnosis, medication and outcome scores.*

***MRS**

TABLE 2: Modified Rankin Scale for Children and Adults

Score	Children	Young Adults
0	No symptoms at all	No symptoms at all
1	No significant disabilities despite symptoms; behavior appropriate to age and normal further development	No significant disability despite symptoms; able to carry out all usual duties and activities
2	Slight disability; unable to carry out all previous activities, but same independence as other age- and sex-matched children (no reduction of levels on the gross motor function scale)[28]	Slight disability; unable to carry out all previous activities, but able to look after own affairs without assistance
3	Moderate disability; requiring some help, but able to walk without assistance; in younger patients adequate motor development despite mild functional impairment (reduction of 1 level on the gross motor function scale)	Moderate disability; requiring some help, but able to walk without assistance
4	Moderately severe disability; unable to walk without assistance; in younger patients reduction of at least 2 levels on the gross motor function scale	Moderately severe disability; unable to walk without assistance and unable to attend to own bodily needs without assistance
5	Severe disability; bedridden, requiring constant nursing care and attention	Severe disability; bedridden, incontinent and requiring constant nursing care and attention
6	Dead	Dead

REF: Bigi S, Fischer U, Wehrli E, *et al.* Acute Ischemic Stroke in Children versus Young Adults. Ann Neurol 2011;70(2):245-54.

****PCPC**

Table I. Pediatric Cerebral Performance Category Scale

Score	Category	Description
1	Normal	Normal; at age-appropriate level; school-age child attending regular school classroom
2	Mild disability	Conscious, alert, and able to interact at age-appropriate level; school-age child attending regular school classroom but grade perhaps not appropriate for age; possibility of mild neurologic deficit
3	Moderate disability	Conscious; sufficient cerebral function for age-appropriate independent activities of daily life; school-age child attending special education classroom and/or learning deficit present
4	Severe disability	Conscious; dependent on others for daily support because of impaired brain function
5	Coma or vegetative state	Any degree of coma without the presence of all brain death criteria; unawareness, even if awake in appearance, without interaction with environment; cerebral unresponsiveness and no evidence of cortex function (not aroused by verbal stimuli); possibility of some reflexive response, spontaneous eye-opening, and sleep-wake cycles
6	Brain death	Apnea, areflexia, and/or electroencephalographic silence

REF: Fiser DH Outcome evaluations as measures of quality in pediatric intensive care. Pediatr Clin North Am. 1994 Dec;41(6):1423-38.

Thank you for completing this questionnaire.

Please return to Dr Sukhvir Wright (c/o Professor Angela Vincent), Neurosciences Laboratory, West Wing Level 5, John Radcliffe Hospital, Oxford. OX3 9DS. Fax: 01865 234320 Email: sukhvir.wright@nhs.net
